# The effect of explicit and implicit online self-compassion interventions on sleep quality among Chinese adults: A longitudinal and diary study

**DOI:** 10.3389/fpsyg.2023.1062148

**Published:** 2023-02-03

**Authors:** Haili Sheng, Ruibing Wang, Conghui Liu

**Affiliations:** Department of Psychology, Renmin University of China, Beijing, China

**Keywords:** sleep quality, self-compassion intervention, implicit intervention, explicit intervention, daily diary study

## Abstract

**Objective:**

This study aimed to investigate the effects of explicit and implicit online intervention methods for self-compassion on improving sleep quality among Chinese adults.

**Methods:**

A total of 328 adult participants were recruited to complete the Pittsburgh Sleep Quality Questionnaire and Self-compassion Scale, and 168 participants were randomly assigned to one of three different conditions: two self-compassion intervention groups (self-compassion writing intervention asked participants to write several sentences with self-compassion, and self-compassion combination intervention asked participants to combine words into complete sentences with self-compassion) and one control group. After 1 week of online self-compassion intervention with daily sleep quality measured simultaneously, 150 participants completed the posttest of the Pittsburgh Sleep Quality Questionnaire and Self-Compassion Scale.

**Results:**

The pretest and posttests results showed that the self-compassion level and sleep quality of the self-compassion writing intervention group were significantly better than those of the control group. However, there was no significant difference between the self-compassion combination intervention group and the control group. For the diary tracking results, sleep quality was significantly better for both the self-compassion writing intervention group and self-compassion combination intervention group than the control group, however, the self-compassion writing intervention group showed great improvements.

**Conclusion:**

Both self-compassion writing and combination interventions were effective in improving sleep quality, and the effect of self-compassion writing was more stable.

## Introduction

Sleep problem is a general issue that disturbs modern society (Chattu et al., 2018). Multiple studies have found that poor sleep is associated with many negative health outcomes, including cognitive impairment (Killgore, 2010; Bubu et al., 2017), mood disorders (Hertenstein et al., 2019; Palagini et al., 2022), non-adaptive behaviors (Lissak, 2018), increased risk of disease (Irwin, 2015) and death (Vaccaro et al., 2020; Heilbrunn et al., 2021). Although medication is a common treatment for sleep disorders, it can cause side effects. For example, medications for insomnia may cause drowsiness and dizziness (Qaseem et al., 2016). This shifts our attention to psychological factors that can improve sleep quality. Emerging studies have shown that self-compassion protects against sleep problems (Kemper et al., 2015; Sirois et al., 2015; Hu et al., 2018; Kim and Ko, 2018; Butz and Stahlberg, 2020; Rakhimov et al., 2022; Semenchuk et al., 2022). A meta-analysis of the association between self-compassion and sleep quality showed that individuals with higher self-compassion reported fewer sleep problems (Brown et al., 2021). Brief interventions on self-compassion before bed can improve sleep quality (Butz and Stahlberg, 2018; Hu et al., 2018).

Self-compassion refers to individuals being open to their suffering, understanding and having a non-judgmental attitude toward their own inadequacies and failures, and recognizing that their experiences are part of the common human experience (Neff, 2003b). Multiple studies have shown that self-compassion interventions can improve sleep quality (Wong and Mak, 2016; Butz and Stahlberg, 2018; Hu et al., 2018). Most intervention studies on self-compassion intervention for nonclinical populations use the 8-week Mindful Self-Compassion (MSC) training developed by Neff and Germer (2013) or make adjustments based on it (Smeets et al., 2014; Bluth et al., 2016; Bluth and Eisenlohr-Moul, 2017; Campo et al., 2017; Dundas et al., 2017; Finlay-Jones et al., 2017; Neff et al., 2020). Participation in MSC significantly increases self-compassion, mindfulness, and life satisfaction and reduces emotional problems such as depression and anxiety (Neff and Germer, 2013; Neff, 2022). However, because MSC is time intensive and involves meditation training, it requires a high professional level of mentoring (Neff et al., 2020). Researchers have developed more efficient interventions to adapt to the rapid pace of modern life and the needs of different populations (Galla, 2016; Wong and Mak, 2016; Dundas et al., 2017; Finlay-Jones et al., 2017; Neff et al., 2020; Seekis et al., 2020). Meanwhile, interventions have been developed to be convenient for online training (Lennard et al., 2021; Nadeau et al., 2021; Sawdon et al., 2021). Based on recent online psychological counseling and intervention practices, we speculated that simplified online self-compassion practice will enhance self-compassion and sleep quality.

Regarding methods of self-compassion intervention, previous studies have mostly adopted methods that require participants to consciously complete self-compassion exercises, such as letter writing, diary writing, and meditation training (Wong and Mak, 2016; Finlay-Jones et al., 2017; Stevenson et al., 2019). Such intervention methods for inducing conscientious behaviors in participants enable them to acquire knowledge through active thinking and practice, which is a method of explicit process. In addition to conscious interventions, the health models of behavior change involve implicit processes (Sheeran et al., 2013). Recently, the rise of the application of the dual-process theory in health psychology reflects an interest in how implicit processes affect health behavior, and also leads us to focus on the effects of implicit interventions on self-compassion (Hagger, 2016). Dual-process theory suggests that human behavior is influenced by two processes: a conscious, deliberative process and an unconscious, implicit process (Kahneman, 2011; Hagger, 2016). Considering the popular models of cognition (e.g., TPB target) sometimes have difficulty explaining and changing behavior (Sniehotta et al., 2014), the ability to self-regulate and thus change attitude and behavior may depend on implicit processes (St Quinton and Brunton, 2017). For example, in the study of Levy et al. (2014), although both implicit and explicit intervention for older adults significantly strengthened positive age stereotypes, only implicit intervention further improved physical function.

Implicit and explicit intervention methods offer several advantages. According to previous research on learning letter strings, language, etc., it is advantageous to learn concise languages and thinking patterns through explicit and subjective efforts (Wang, 2020). However, it is possible for an explicit intervention to be hindered by pre-existing beliefs. Implicit intervention has strong generality and abstraction and has advantages in many aspects, such as internal structure learning, which requires abstract processing (Wang, 2020). Although implicit processes are included in some behavior change theories, effective interventions are still scarce and need further development (Hollands et al., 2016). Implicit interventions may work by strengthening associations between stimuli (Hollands et al., 2016), so that implicit interventions on self-compassion may cause participants to unconsciously associate themselves with high self-compassion. To date, no study has explored self-compassion interventions from an implicit perspective. Therefore, this study also aimed to explore the effects of self-compassion intervention through implicit learning.

This study examined whether these two intervention methods could improve self-compassion, thereby improving sleep quality. One is the traditional method of self-compassion writing (Sherman et al., 2018; Krieger et al., 2019; Stevenson et al., 2019; Schnepper et al., 2020; Seekis et al., 2020), which allows participants to actively recall, imagine, and experience events, and then write down their thoughts. The other is the self-compassion combination method, which we developed based on the implicit learning theory (Dweck et al., 1995). This method does not specify the intervention purpose in the instructions and questions; instead, it asks participants to integrate phrases into sentences that evoke self-compassion, thus improving self-compassion and sleep quality. Our data were collected based on a longitudinal study and diary method. We hypothesized that both the self-compassion writing method and self-compassion combination method would significantly improve self-compassion and therefore sleep quality. Considering that self-compassion entails clear language and thought patterns (Neff, 2003b), we hypothesized that the intervention effect of explicit self-compassion writing was better than that of implicit self-compassion combination.

## Methods

### Study design and participants

A longitudinal study with a time interval of 1 week and a 7-day diary survey was conducted to test the hypotheses. According to G*Power 3.1, 159 samples were required to predict a statistical power level of 80% with a significance level of *α* = 0.05 and an effect size of *f* = 0.25. In total, 328 participants were recruited to complete the pretest questionnaire, 311 of which were valid, with an effective response rate of 94.8%. At least 199 participants agreed to complete the 1-week diary survey and posttest. Of these, 168 completed the pretest and the daily questionnaire for more than 2 days (effective completion rate was 82%; that is, each participant completed the daily follow-up questionnaire 5.8 times on average), and 150 completed the final posttest, including 39 in the self-compassion writing intervention group, 43 in the self-compassion combination intervention group, and 68 in the control group. The demographic information of the three groups is presented in Table 1.

**Table 1:** Sample distribution of the three groups (*N* = 150).

	Self-compassion writing intervention group		Self-compassion combination intervention group		Control group		Full sample	
	*n*	%	*n*	%	*n*	%	*n*	%
Gender								
Male	9	6.0	10	6.7	14	9.3	33	22.0
Female	30	20.0	33	22.0	54	36.0	117	78.0
Age								
Under 20	0	–	0	–	3	2.0	3	2.0
20–29	5	3.3	8	5.3	12	8.0	25	16.7
30–39	11	7.3	10	6.7	17	11.3	38	25.3
40–49	19	12.7	19	12.7	30	20.0	68	45.3
50–59	4	2.7	6	4.0	6	4.0	16	10.7

### Measurement

#### Pittsburgh sleep quality index

The Pittsburgh Sleep Quality Index (PSQI) was used to measure self-reported sleep quality of individuals (Buysse et al., 1989). It includes 19 items that are divided into seven dimensions. Each dimension was scored on a scale ranging from 0 to 3. The higher the score, the worse the sleep quality. The PSQI has good reliability and validity in both clinical and nonclinical samples (Takács et al., 2016). In this study, Cronbach’s *α* for the PSQI was 0.75.

#### Self-compassion scale

The Chinese version of the Self-Compassion Scale (SCS) was used to assess self-compassion (Neff, 2003a; Chen et al., 2011). The scale consisted of 26 items divided into six dimensions. Each item on this scale ranges from 1 to 7. The three dimensions were scored positively, whereas the others were scored inversely. A high total score indicates a high level of self-compassion. In this study, Cronbach’s *α* for the scale was 0.89.

#### The daily questionnaire

The daily questionnaire included PSQI and self-compassion intervention questions. The “last month” in the instruction of PSQI was adjusted to “last night.” The subscale “sleep latency” calculated the length of the time between when one goes to bed and falls asleep, and “sleep duration” calculated the time between falling asleep and getting up. In order to reduce the number of questions that participants needed to answer, the direct question asking about sleep time was removed. The questions asking how often sleep problems (e.g., difficulty falling asleep) occur was changed to “the number of times you woke up during sleep in last night.” Self-compassion intervention questions were used for each group.

#### Procedures

Recruitment information was sent through WeChat Moments (a phone app) using a convenience sampling method to introduce the research topic to potential participants. On August 4, 2021, participants who understood the experiment content and gave informed consent completed pretest questionnaires, including the Pittsburgh Sleep Quality Index and the Self-Compassion Scale. They were then divided into one of three groups: self-compassion writing intervention group, self-compassion combination intervention group, and control group, according to the end of their telephone number. The intervention period was August 5–11. During the intervention period, participants in each group were invited to join the WeChat group and a questionnaire was sent through WeChat every day. Participants were asked to complete daily self-compassion intervention questions. Participants in the self-compassion writing intervention group were asked to complete a daily writing task. The topics were chosen from *Self-Compassion: Stop Beating Yourself Up and Leave Insecurity Behind*, edited by Neff and transformed by Neff (2012). For example, “Think about one or two of the most challenging events in your life. At the time, the difficulties faced were overwhelming. Now, with the benefit of hindsight, can you think of something good that came out of that?” The participants in the self-compassion combination intervention group completed three ordering tasks each day, which were selected from positive items in the Self-Compassion Scale, such as “please organize the following words into complete sentences: kind to myself, I am, suffering, when I’m experiencing.” The control group did not receive any intervention questions. Simultaneously, participants completed the adjusted PSQI according to the sleep state of the previous day. The daily questionnaire of the day was sent out through a WeChat group at 7 a.m. every day, and participants could fill out the questionnaire at any time during the day. After seven consecutive days of intervention questionnaires, participants were asked to complete post-test questionnaires. Participants were paid 1 yuan (approximately $0.14) daily to complete the questionnaire. All procedures performed in studies involving human participants were in accordance with the ethical standards of the institutional and national research committee and with the 1964 Helsinki Declaration and its later amendments.

### Statistical analysis

The data were analyzed using SPSS 25.0. Correlation and multivariate analyses of variance were used to test the relationship between self-compassion and sleep quality and the effects of different self-compassion interventions on sleep quality.

## Results

### The differences in self-compassion and sleep quality among different groups

A correlation analysis of 311 pretest data showed that self-compassion was negatively correlated with the sleep quality index (*r* = −0.19, *p* < 0.001), indicating that higher self-compassion scores were associated with better sleep quality.

To evaluate the differences after a 7-day intervention among the self-compassion writing intervention group, self-compassion combination intervention group, and control group, the differences in sleep quality and self-compassion between pretest and posttest scores were calculated, and the differences were analyzed by a one-way analysis of variance (ANOVA). The correlations between the differences in sleep quality and self-compassion and demographic variables (including age, sex, type of career, and education) were calculated, and no significant correlation was found. Therefore, no control variables were included in the subsequent analyses. As shown in Table 2, there were significant differences in the mean scores for sleep quality and self-compassion among the groups. According to the LSD multiple comparisons, compared to the control group, sleep quality (*p* = 0.002) and self-compassion (*p* = 0.016) were significantly improved in the self-compassion writing intervention group. Sleep quality (*p* = 0.510) and self-compassion (*p* = 0.615) were also improved in the self-compassion combination intervention group, but the differences were not significant.

**Table 2:** The difference in self-compassion and sleep quality between pretest and posttest (*M* ± *SD*).

Outcomes		SWIG	SCIG	CG	*F*	*p*	*η*_p_^2^
Self-compassion	Pretest	3.48 ± 0.53	3.49 ± 0.46	3.41 ± 0.51	0.39	0.677	0.01
	Posttest	3.70 ± 0.60	3.57 ± 0.55	3.45 ± 0.58	2.33	0.101	0.03
	Difference	0.22 ± 0.41	0.08 ± 0.38	0.04 ± 0.34	3.09	0.048	0.04
Sleep quality	Pretest	0.86 ± 0.46	0.71 ± 0.28	0.88 ± 0.43	2.69	0.071	0.04
	Posttest	0.67 ± 0.39	0.68 ± 0.29	0.90 ± 0.40	6.78	0.002	0.08
	Difference	−0.19 ± 0.33	−0.02 ± 0.27	0.02 ± 0.36	5.04	0.008	0.06

SWIG, self-compassion writing intervention group; SCIG, self-compassion combination intervention group; CG, control group; Difference, difference between pretest and posttest.

### Diary tracking analysis of different interventions

To explore the sleep quality fluctuations of participants in different groups during the self-compassion intervention, 18 participants who did not complete the posttest but completed the pretest and the daily questionnaire for more than 2 days were included in the analysis. Thus a total of 168 participants were included in this part of analyses. A repeated measures ANOVA was conducted with intervention methods (self-compassion writing intervention group, self-compassion combination intervention group, control group), and time (Wednesday, Thursday, Friday, Saturday, Sunday, Monday, and Tuesday) as independent variables and sleep quality as the dependent variable. The results showed that the main effect of the intervention method was significant [*F*(2, 165) = 3.36, *p* = 0.037, *η_p_*^2^ = 0.04]. According to pairwise comparisons, there were significant differences between the self-compassion writing intervention group and the control group (*p* = 0.043) and between the self-compassion combination intervention group and the control group (*p* = 0.025). The main effect of time was not significant [*F*(6, 990) = 0.65, *p* = 0.691, *η_p_*^2^ = 0.02]. The interaction effect between intervention and time was significant [*F*(12, 990) = 1.83, *p* = 0.043, *η_p_*^2^ = 0.06]. A further simple effect analysis revealed that the main effects of the intervention methods were significant on Saturday (*p* = 0.035) and Tuesday (*p* < 0.001), but not on Wednesday (*p* = 0.287), Thursday (*p* = 0.472), Friday (*p* = 0.617), Sunday (*p* = 0.174), and Monday (*p* = 0.523).

To explain the differences between the two intervention groups and the control group in more detail, we analyzed the differences in sleep quality improvement (Day7 − Day 1). The result of one-way ANOVA showed a significant difference in the sleep quality improvement among the three groups, *F*(2, 165) = 3.33, *p* = 0.038, *η_p_*^2^ = 0.04. LSD *post-hoc* test showed that sleep quality improvement in the self-compassion writing intervention group (Day7 − Day 1 = −0.52, *p* = 0.025) and self-compassion combination intervention group (Day7 − Day 1 = −0.38, *p* = 0.044) was significantly better than that of the control group (Day7 − Day 1 = 0.52). There was no significant difference between the self-compassion writing intervention group and the self-compassion combination intervention group (*p* = 0.777). The results showed that the effect of the intervention on self-compassion gradually improved sleep quality over the week, and that both intervention methods showed significant improvement.

Moreover, we conducted an additional analysis of the time spent on the daily tracking questionnaire for the three groups of participants. One-way ANOVA revealed significant differences in time spent by the three groups, *F*(2, 165) = 15.34, *p* < 0.001. LSD *post-hoc* test revealed significant differences in time spent between the self-compassion writing group (the average time was 370 s) and the self-compassion combination intervention group (251 s), *p* < 0.001. The differences between the self-compassion writing group and the control group (208 s) were also significant, *p* < 0.001. The differences in time spent between the self-compassion combination intervention group and the control group were not significant, *p* = 0.112.

## Discussion

The results of this study showed a significant negative correlation between self-compassion and the sleep quality index. Simplified online self-compassion training for 1 week could effectively improve the level of self-compassion and sleep quality. The effect of the explicit subjective writing exercise was more stable, as the difference between the pretest and posttest was significant, as was the result of the diary tracing analysis.

First, from the results of the intervention, the 1-week online self-compassion training significantly improved self-compassion and sleep quality in the intervention group. This result was consistent with previous studies on the improvement of sleep quality by self-compassion intervention in the form of written letters (Neff, 2003a; Wong and Mak, 2016; Hu et al., 2018), on the basis that the writing topic was simplified compared to previous studies (Neff, 2012; Neff and Germer, 2013). Research has shown that the improvement of self-compassion helps individuals objectively understand their problems, treat themselves well, actively seek their own internal understanding and comfort, and recognize their experiences as shared experiences with other human beings (Neff, 2003a,b). Individuals with higher levels of self-compassion can perceive their situation in a more balanced and present-focused manner; therefore, they are more likely to have fewer negative emotions and improve their sleep quality (Neff, 2003a,b; Finlay-Jones, 2017; Inwood and Ferrari, 2018).

Second, comparing the effects of self-compassion writing and self-compassion combination, explicit subjective writing had a greater effect on self-compassion and sleep quality, while implicit objective combination only showed a significant improvement in sleep quality in the diary tracking analysis. This difference may be related to the scope of the application of different learning methods (Wang, 2020). For example, implicit learning is suitable for learning with high abstractness and difficulty level. However, explicit learning is suitable for learning content that is less abstract, more intuitive, and requires individual subjective effort. Another possible explanation is that participants in the two intervention groups spent different amounts of time completing the self-compassion activities. Even though the questionnaire was designed to account for the balance of time, the self-compassion writing intervention group still spent an average of 34 s more on thinking and writing than the self-compassion combination intervention group each day, which might contribute to a better intervention. Third, the results may be affected by data properties. A previous study showed that compared with standardized scales or objective instruments, dynamic diaries may more accurately record the daily state of participants than estimate it based on long-term memory (Barnes et al., 2013). Therefore, the results of the 7-day diary follow-up analysis may be more accurate than the results of the comparison of pre-and posttest differences. In practice, implicit and explicit learning methods can be used together to promote the practice effect (Santiago and Axel, 2020), so the integration of subjective writing and objective combination may have a better effect.

These findings should be interpreted in the context of certain limitations. First, although we used a randomized method to construct three equal groups, there were certain differences in the initial levels of self-compassion and sleep quality among the three groups. Therefore, we did not choose the original value but used the difference values of the pretest and posttest for the analysis of variance. At the same time, the repeated measures ANOVA faces the problem of insufficient sample size, which contains data from 150 participants. However, as stated in the method section, a sample size of 159 would yield sufficient power. Future studies should use larger samples to balance the experimental and control groups. Second, the control group lacked a comparable control task. Future studies should have an appropriate control task for the control group. Third, the intervention time of this study was relatively short, and discussion on the size and stability of the intervention effect requires studies with longer interventions (Dundas et al., 2017; Mistretta and Davis, 2021). Fourth, although the PSQI is an effective and widely used tool for measuring sleep quality (Carpenter and Andrykowski, 1998; Brown et al., 2021), it relies on self-reported results. As subjective and objective sleep measures capture different aspects of sleep that are independently related to functional status (Teas and Friedman, 2021), future research could combine self-report questionnaires with objective measures (e.g., EEG and EMG). Fifth, the end number of the phone number was used to divide the participants into the experiment group and the control group, which may indeed have the problem of insufficient randomness. For example, in Chinese culture, people usually believe that the number 8 is lucky. However, when we excluded participants with phone numbers ending in 8, there was no significant change. So, the effect of this distribution on the results is likely to be small. Finally, the study only focused on the effects of different interventions for self-compassion on sleep quality, and the internal mediating mechanisms need to be studied further (Semenchuk et al., 2022). The influence of a week’s effect on the outcome of the intervention can also be explored further (Ul Ain et al., 2021).

## Conclusion

In conclusion, this study attempted to find efficient sleep improvement methods to adapt to fragmented life and provide empirical exploration for simplified online self-compassion interventions. This study verified the effect of explicit and implicit self-compassion interventions; both had significant improvement effects on sleep quality, which is likely to provide a new perspective and enlightenment for future intervention research.

## Data availability statement

The raw data supporting the conclusions of this article will be made available by the authors, without undue reservation.

## Ethics statement

The studies involving human participants were reviewed and approved by the Institutional Review Board at Renmin University of China. The patients/participants provided their written informed consent to participate in this study.

## Author contributions

HS contributed to the design of methodology, data collection and analysis, and preparation of the initial draft. RW contributed to the preparation, creation, and presentation of the published work. CL contributed to the formulation and evolution of the research goals and aims. All authors contributed to the article and approved the submitted version.

## Funding

This work was supported by the Fundamental Research Funds for the Central Universities, and the Research Funds of Renmin University of China (20XNA028).

## Conflict of interest

The authors declare that the research was conducted in the absence of any commercial or financial relationships that could be construed as a potential conflict of interest.

## Publisher’s note

All claims expressed in this article are solely those of the authors and do not necessarily represent those of their affiliated organizations, or those of the publisher, the editors and the reviewers. Any product that may be evaluated in this article, or claim that may be made by its manufacturer, is not guaranteed or endorsed by the publisher.
